# Combining high-throughput imaging flow cytometry and deep learning for efficient species and life-cycle stage identification of phytoplankton

**DOI:** 10.1186/s12898-018-0209-5

**Published:** 2018-12-03

**Authors:** Susanne Dunker, David Boho, Jana Wäldchen, Patrick Mäder

**Affiliations:** 1 Department of Physiological Diversity, Helmholtz-Centre for Environmental Research-UFZ, Permoserstraße 15, 04318 Leipzig, Germany; 2 Department of Physiological Diversity, German Centre for Integrative Biodiversity Research-iDiv, Deutscher Platz 5a, 04103 Leipzig, Germany; 3 Software Engineering for Safety-Critical Systems Group, Technische Universität Ilmenau, Ehrenbergstraße 29, 98693 Ilmenau, Germany; 4 Department of Biochemical Integration, Max-Planck-Institute for Biogeochemistry, Hans-Knöll-Straße 10, 07745 Jena, Germany

**Keywords:** Imaging flow cytometry, Phytoplankton, Morphology, Deep learning, CNN, Images, Image-based identification, Machine learning, High throughput cytometry, Magnification

## Abstract

**Background:**

Phytoplankton species identification and counting is a crucial step of water quality assessment. Especially drinking water reservoirs, bathing and ballast water need to be regularly monitored for harmful species. In times of multiple environmental threats like eutrophication, climate warming and introduction of invasive species more intensive monitoring would be helpful to develop adequate measures. However, traditional methods such as microscopic counting by experts or high throughput flow cytometry based on scattering and fluorescence signals are either too time-consuming or inaccurate for species identification tasks. The combination of high qualitative microscopy with high throughput and latest development in machine learning techniques can overcome this hurdle.

**Results:**

In this study, image based cytometry was used to collect ~ 47,000 images for brightfield and Chl *a* fluorescence at 60× magnification for nine common freshwater species of nano- and micro-phytoplankton. A deep neuronal network trained on these images was applied to identify the species and the corresponding life cycle stage during the batch cultivation. The results show the high potential of this approach, where species identity and their respective life cycle stage could be predicted with a high accuracy of 97%.

**Conclusions:**

These findings could pave the way for reliable and fast phytoplankton species determination of indicator species as a crucial step in water quality assessment.

## Background

Phytoplankton monitoring is a crucial part of the biological water quality assessment [30, 47]. Regular monitoring of indicator taxa reveals potential impairment of water life in general and specifically for human usage [11, 35, 53]. The traditional standard of phytoplankton analysis for monitoring is microscopy, being time-consuming and requiring taxonomic expertise [5, 6, 11, 17, 18, 30]. High species diversity and detritus complicate the acquisition of the typically required minimum of 500 cells [30] often resulting in a multi-hour counting process for the expert [18, 33]. Due to this long analysis time, it is necessary to fix samples leading to artifacts and a loss of pigments, which could be otherwise helpful for species identification. Culverhouse et al. [13], Embleton et al. [17] and First and Drake [18] investigated the accuracy and reliability of human microscopic counting of species. First and Drake [18] asked experts with a minimum working experience greater than 7 years to identify species. The time span, selected by the authors, demonstrates how many years of expertise are thought to be necessary to guarantee reliable measurements of natural phytoplankton communities. Hofstraat et al. [28] state that taxonomy experts are expected to have quantification errors of ~ 10% for dominant species and even ~ 60% for rare species. Culverhouse [11] define four factors that limit the human performance in species identification: short-term memory of five to nine items, boredom and fatigue, recency effects and positivity bias. Furthermore, a shortage of taxonomic expertise is expected in the near future [12].

The demand for experts and the time-consuming species identification process strongly limit the feasible number of samples that can be analyzed. At the same time, there is demand for more frequent sampling, since species show high growth rates and are sensitive to environmental change, meaning that there is a high temporal and spatial variance of phytoplankton abundance [35, 48], especially with respect to human activities like eutrophication, climate change and introduction of invasive species. Automating this task is highly desirable, especially considering the continuous loss of experienced taxonomists with the simultaneous requirements for increased environmental monitoring.

As an alternative to traditional microscopy, analytical flow cytometers (AFC) were used for species identification [1, 5, 6, 19, 52]. AFC can process thousands of cells per second, making this analysis substantially faster than microscopy and enabling higher sampling frequencies [39]. AFC uses hydrodynamically focused cells in a flowing sheath stream and laser excitation to measure the corresponding scatter and several emission signals. AFC guarantees high throughput of the organisms on an individual base level. However, this technique never became widely established for species identification; instead microscopy is still common practice [18].

**Fig. 1:**
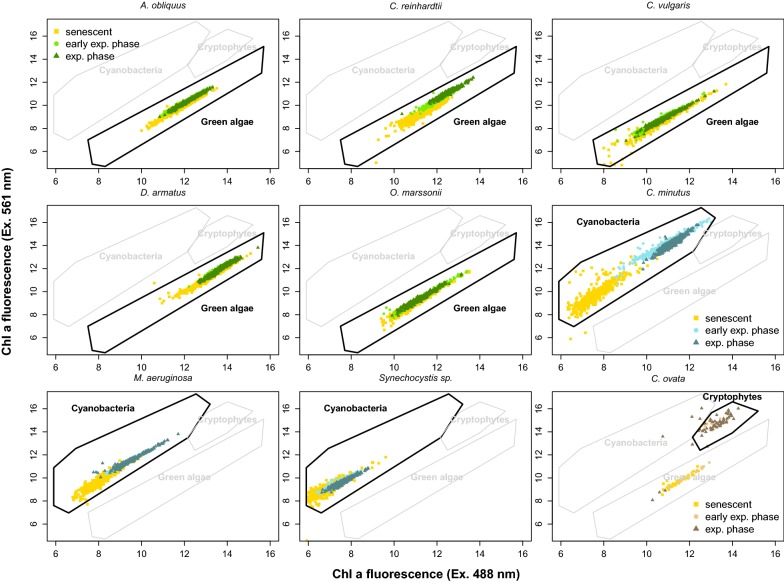
Demonstration of variability of fluorescence pattern depending on different growth stages during early exponential, exponential and stationary phase for all nine species. Presented as Chl *a* fluorescence excited by a 488 nm (x-axis) and a 561 nm laser excitation (y-axis). Yellow dots represent senescent cells during stationary phase, light green, blue or brown dots represent cells growing in early exponential phase and green, blue or brown dots represent cell growing in exponential phase

AFC allows a quantitative analysis of cell abundance and extraction of several individual-level parameters, like the Forward Scatter signal (indicative for cell size), the Sideward Scatter signal (indicative for granularity) and fluorescence emission values, resulting from different laser excitation, scatter and fluorescence emission channels. Scatter and fluorescence values are used to prepare two-dimensional plots, where point clouds appear [7, 33]. A high accumulation of points helps to define potential populations or sub-populations of species (gating). But the setting of gates to define a population or sub-population is subjective and strongly dependent on the expertise of the operator [7, 33]. There are two possible ways from two-dimensional point clouds or principal component clouds of multidimensional data to species identification: (1) sorting of fractions with subsequent manual inspection, and (2) using pigment signatures from pure laboratory grown cultures. Many species of the same taxonomic group typically share a similar pigmentation letting their point clouds overlap. A sorted fraction of a field sample contains many different species making it impossible to uniquely assign one sorted cell to a single species one to one. Ironically, this manual inspection by sorting is similarly time-consuming as the microscopic approach. In contrast, species assignment via fluorescence pattern of pure cultures is inaccurate and extremely difficult or almost impossible to apply to field measurements, because the fluorescence emission pattern of the same species grown in the laboratory and that grown in nature could be totally different [1, 52]. This divergence is mainly caused by differences in natural light and nutrient conditions. Phytoplankton species show a plastic response of pigmentation to be optimally acclimatized to environmental conditions [10], meaning that Chl *a* content per organic matter varies in a large range of 0.1–5% [21, 22]. In this study, we created variation of Chl *a*: biomass by using samples at different life cycle stages (Fig. 1).

Several authors studied machine learning techniques to improve species identification from AFC data [1, 6, 19, 50–52]. Although the identification accuracy of these approaches is often promising, scatter properties and fluorescence emission data taken in the laboratory are not easily transferable to field samples as already mentioned. This means that calibrating identification classifier like neural networks with AFC data from laboratory samples and applying these on field samples is therefore highly erroneous [52]. This means that in practice indicator taxa can often not be uniquely identified using this approach.

Despite obvious benefits of AFC with regard to measuring speed, major drawbacks are a limited taxonomic resolution at the species level and low information contents of single scatter or fluorescence values [8, 27].

To overcome the mentioned limitations of the microscopic count and AFC approaches the use of imaging flow cytometry (IFC) in combination with latest computer vision techniques seems to be promising. IFC, a hybrid technology combining speed and statistical capabilities of flow cytometry with imaging features of microscopy, is rapidly advancing as a cell imaging platform that overcomes many of the limitations of current and previous techniques. Different devices are comprehensively reviewed by Dashkova et al. [14]. Using images for automated species identification has the advantage that images contain the same information that also a taxonomist would use for species identification, i.e. size, form, internal structures and conspicuous features, but are sampled much faster and being substantially richer than highly aggregated scatter or fluorescence signals, which do not contain sub-cellular fluorescence localization [2, 26]. Furthermore, morphological properties, e.g. cell volume of species, are much less impacted by variation of environmental conditions than Chl *a* content per cell [20] and are therefore more robust for species identification.

Similar to automated analysis of scatter and fluorescence signals, a number of successful approaches have been proposed for automated analysis of phytoplankton images [4, 8, 17, 23, 31, 37, 42, 45]. Many approaches to classify species from images are based on previously extracted features, such as diameter, volume or aspect ratio of the organisms. Here, the feature selection was a critical step in designing an optimal phytoplankton classification system. Deriving highly informative and complementary features is essential for high classification performance, but the process is labor-intensive, requires domain knowledge and is often subjective. Deep artificial neural networks (CNN) automate these critical feature extraction steps by learning a suitable representation of the training data and by systematically developing a robust classification model [49]. CNNs are increasingly used in imaged based phytoplankton identification [31, 37]. However, a full automation of microscopic phytoplankton species measurement in combination with CNN was not shown yet. Furthermore, analyzing and predicting life stages were neglected although it reveals important additional information about the physiological state of the phytoplankton species.

The overall aim of this study is (a) to show whether species of nano- and microplankton can automatically be identified using deep neuronal networks, (b) to evaluate whether also their life cycle stage can be identified, and (c) to determine the most appropriate combination of available image channels for (a) and (b).

## Material and methods

### Species

Tested species [*Acutodesmus obliquus* (SAG 276-3a, formerly called: *Scenedesmus obliquus* (Turpin) Kützing, *S. acutus f. alternans*), *Chlamydomonas reinhardtii* (SAG 11-32b), *Chlorella vulgaris* (SAG 211-11b), *Chroococcus minutus* (SAG 41.79), *Cryptomonas ovata* (SAG 979-3), *Desmodesmus armatus* (SAG 276-4 d, formerly called: *Scenedesmus quadricauda*, *Scenedesmus armatus* Chodat), *Microcystis aeruginosa* (SAG 1450-1), *Oocystis marssonii* (SAG 257-1) and *Synechocystis* sp. (PCC 6803)] (overview in Table 1) were purchased from Culture Collection of Algae EPSAG (Göttingen, Germany) and Pasteur Culture Collection (Paris, France), following the Microbial Resource Research Infrastructure (MIRRI) Best Practice Manual on Access and Benefit Sharing. All strains were cultivated in different media for 144 days (Bold’s Basal Medium for green algae and the cryptophyte according to Bischoff [3]: NaNO_3_ = 250 mg L^−1^, CaCl_2_^·^7H_2_O = 25 mg L^−1^, MgSO_4_^·^7H_2_O = 75 mg L^−1^, KH_2_PO_4_ = 175 mg L^−1^, K_2_HPO_4_^·^3H_2_O = 98 mg L^−1^, NaCl = 25 mg L^−1^, Fe-EDTA = 1 mL L^−1^, micronutrient solution = 2 mL L^−1^ and Zehnder-Medium for cyanobacteria according to Staub [46]: NaNO_3_ = 467 mg L^−1^, Ca(NO_3_)_2_^·^4 H_2_O = 59 mg L^−1^, MgSO_4_^·^7H_2_O = 25 mg L^−1^, K_2_HPO_4_^·^3H_2_O = 31 mg L^−1^, Na_2_CO_3_ = 21 mg L^−1^, Fe-EDTA-complex = 10 mL L^−1^, micronutrient solution = 0.08 mL L^−1^) in a 14/10 light/dark cycle on shaking tables with a light intensity of 80 µmol photons m^−2^ s^−1^ and 20 °C in batch culture. At different time points early exponential (day 9 after inoculation), exponential (day 23 after inoculation) and stationary phase (day 144 after inoculation). All species were selected to be (1) common freshwater species, (2) growing under meso-to eutrophic conditions (http://www.algaebase.org, [38], Table 1) and (3) similar in morphology and size range to exhaust the limits of species classification. According to Reynolds [41] and Palmer [36] some of the selected species belong to indicator genera (*A. obliquus* and *D. armatus* formerly called *Scenedesmus*), *Chlamydomonas*, *Chlorella*, *Cryptomonas*, *Microcystis* and *Oocystis* and are marked in Table 1 accordingly. In addition *M. aeruginosa* is a common harmful bloom forming species.

**Table 1:** Overview about investigated species for strain identity, culture medium, cell size and weighted average tolerated Total phosphorus range from a global dataset (according to Phillips et al. [38], Supplementary material)

	Strain	Taxonomic group	Medium	TP-range (µg L^−1^)^a^
*Acutodesmus obliquus* ^b,c^	SAG 276-3a	Green algae	BBM	25–90
*Chlamydomonas reinhardtii* ^b,c^	SAG 11-32b	Green algae	BBM	12–41
*Chlorella vulgaris* ^b,c^	SAG 211-11b	Green algae	BBM	27–87
*Chroococcus minutus*	SAG 41.79	Cyanobacteria	Z-Medium	16–60
*Cryptomonas ovata* ^c^	SAG 979-3	Cryptophyte	BBM	12–41
*Desmodesmus armatus* ^b,c^	SAG 276-4d	Green algae	BBM	25–90
*Microcystis aeruginosa* ^c^	SAG 1450-1	Cyanobacteria	Z-Medium	38–108
*Oocystis marssonii*	SAG 257-1	Green algae	BBM	8–28
*Synechocystis* sp.	PCC 6803	Cyanobacteria	Z-Medium	21–62

^a^Tolerated range of total phosphorus (TP) according to Phillips et al. [38]

^b^Indicator genus according to Palmer [36]

^c^Indicator genus according to Reynolds [41]

### Instrument settings

Phytoplankton samples were measured with a special-order laboratory based imaging flow cytometer ImageStream^®X^ MK II (Amnis part of EMD Millipore, Darmstadt, Germany), able to measure brightfield images and fluorescence images simultaneously. The instrument was chosen to measure nano- and microplankton species by taking images with a high sampling rate at 60× magnification and a comparably good resolution. As basis of an automated routine, many thousands of images were acquired within minutes resulting in sufficient images to train a successful deep learning classifier.

As sheath-fluid Dulbecco’s phosphate buffered saline w/o calcium, w/o magnesium (Biowest, Nuaillé, France) was used. Particles of interest (excluding speed calibration beads) were collected with a 488 nm laser intensity of 0.1 mW. The laser configuration is unique and specifically adapted to the measurement of phytoplankton cells allowing a first separation of different spectral groups and subsequent species identification by morphological information (patent submission PCT/EP2017/075553).

For each species ~ 50 µL of sample were used. Data acquisition was finished, when 5000 events were measured or alternatively when a time of ~ 20 min had elapsed.

All brightfield and Chl *a* fluorescence images (488 nm excitation/642–745 nm emission) were automatically taken at 60× magnification with a numeric aperture of 0.9, a pixel size of 0.3 × 0.3 µm and a 40 × 170 µm field of view. For CNN training, only viable (Chl *a* containing) cells were selected based on a two-dimensional plot of cell area and Chl *a* fluorescence intensity.

### Dataset

**Fig. 2:**
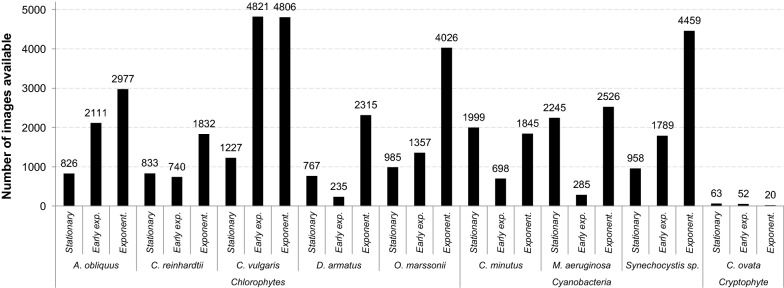
Overview about number of images (brightfield or Chl *a* fluorescence images respectively) included per species and life cycle stage (stationary phase, early exponential phase and exponential phase)

**Fig. 3:**
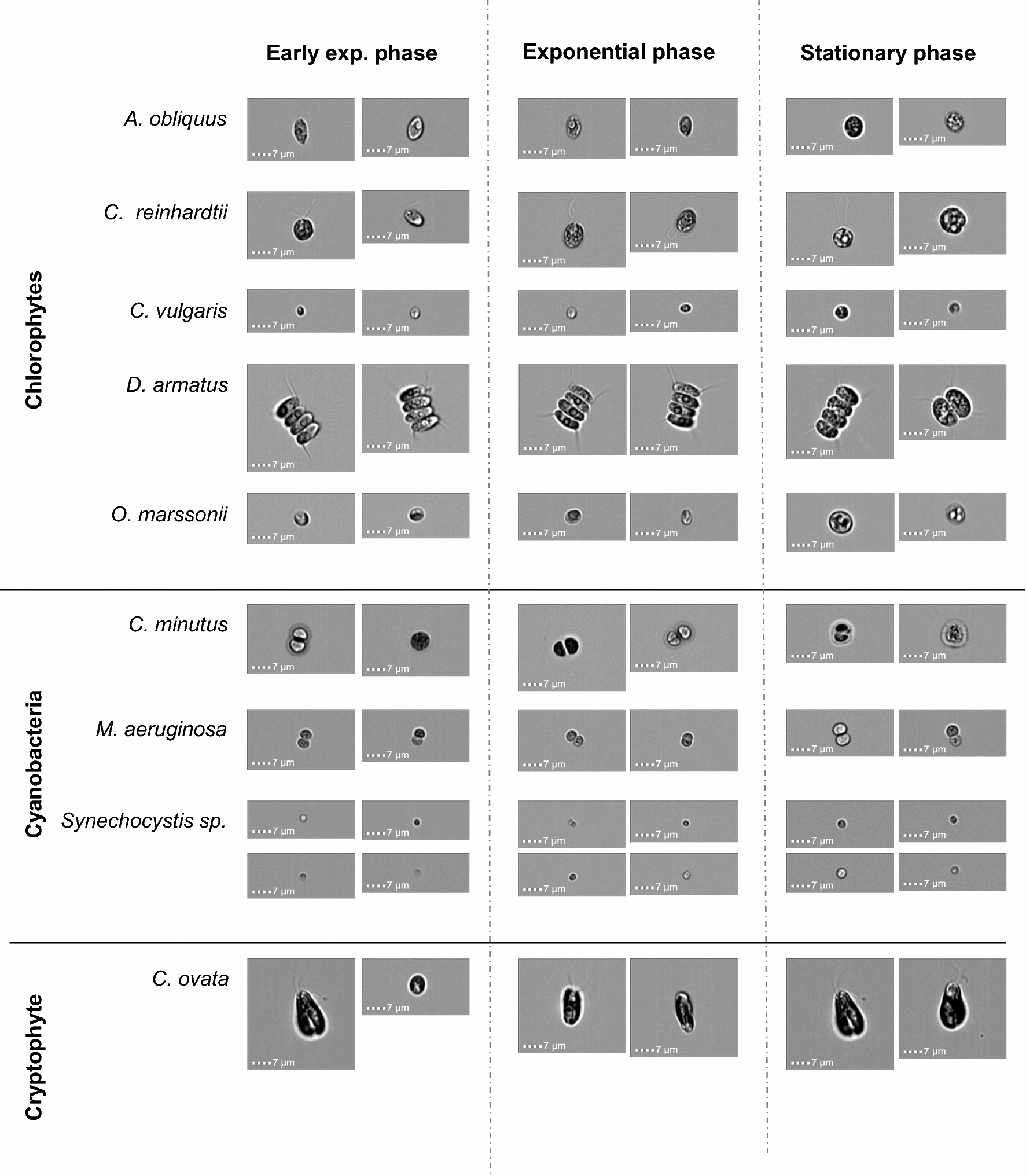
Exemplary brightfield images (two or four images per case) taken with the ImageStream^®X^ MK II (×60 magnification) of each phytoplankton species used in this study for training of the deep learning network at three different life cycle stages (early exponential, exponential and stationary phase)

The dataset consisted of 46,797 brightfield and 46,797 Chl *a* fluorescence images of nine species (Figs. 2 and 3). For each single cell, a brightfield and the respective Chl *a* image was used. Images were collected at three different stages of batch-culture, during early exponential, exponential and stationary phase. For almost all species, most images were available in the exponential phase (Fig. 2).

The complete dataset was split into three sets for training, validation and testing in the proportion 80:10:10. All images were center cropped to an equally sized rectangle. In order to gain a more robust and better generalizing classifier, the images in the training set were additionally augmented in the following ways: flipped horizontal, flipped vertically, adjusted in brightness ± 12.5%, adjusted in saturation ± 50.0%, adjusted in contrast ± 12.5%, and adjusted in hue ± 20%.

### Classifier

As the images taken with the imaging flow cytometer have a lower image quality than a standard microscopic image, it is necessary to take advantage of a powerful deep learning model (classifier) to identify species based on imaging flow cytometric data sets [9]. Therefore, pre-processed images were finally used to train a CNN deep learning model, a common standard deep learning network for visual input. A CNN model uses several layers (input, hidden and output layer) with different image pattern information in a feed-forward mode.

We trained such a CNN classifier on the described training data. More specifically, a residual network architecture (ResNet v2) with 50 convolution layers [24], winning the prestigious ImageNet competition in 2015 and beating for the first time a human performing the same classification task, was used. Transfer learning is a common procedure for training of classifiers with less than ~ 1 M images [54]. That is, we used a network that was pre-trained on the large-scale ImageNet ILSVRC 2012 dataset (http://image-net.org/challenges/LSVRC/2012/) before utilized for our training. Training used a batch size of 32, with a learning rate of 0.0003 and was terminated after 80,000 steps.

In order to assess the characteristic information conveyed per captured image channel in separation and in complementation to each other, we trained four classifiers: (1) brightfield images alone, (2) Chl *a* fluorescence images alone, (3) all brightfield—Chl *a* fluorescence images and (4) merged brightfield—Chl *a* fluorescence images. Classifiers (1) and (2) assess the individual species characteristics conveyed per channel. Classifier (3) is trained with a mixture of images from both channels assessing whether there is complementary information in the channels and representation per class helps in generalizing and creating a more robust classifier. Finally, classifier (4) is trained with two-channel images containing the full amount of available information at training and classification time.

Simultaneously analysis of brightfield and Chl *a* fluorescence images, is expected as best way of classification. Brightfield images contain morphological information, while Chl *a* fluorescence images reveal chloroplast morphology, both being important parameters for traditional taxonomic identification [29]. It is hypothesized that both images carry complementary information helpful for species identification.

#### Validation of classifier performance via confusion matrices

Confusion matrices were prepared to validate classifier performance, including potential species dependent misclassifications in more detail. In the confusion matrices performed, species are shown in rows versus the instances of the same species being predicted in columns. This allows a visualization of how a certain species was confused with others, if its accuracy was below 100% [43].

## Results

### Fluorescence in dependence of life cycle stage

The dataset used in this study, consisted of microscopic images for brightfield and Chl *a* fluorescence, as well as interlinked standard flow cytometric data (fluorescence intensity values) taken with an imaging flow cytometer. To approximately simulate different pigmentation, like it is expected for laboratory cultured and field phytoplankton species, different life cycle stages were used. Figure 1 demonstrates the high variability of cellular fluorescence signals depending on life cycle stage of the batch culture. Besides *C. vulgaris* and *O. marssonii*, all species investigated show strong differences in a two-dimensional fluorescence plot especially between stationary and early exponential/exponential phase. *C. ovata* can even only be identified unambiguously during exponential phase, otherwise phycobilin absorption is so low, that when only fluorescence is used, misidentification with green algae is possible.

### Visual description of the species

An impression of images used to train the CNN is provided in Fig. 3. Most investigated species show similar morphology (coccal: *C. vulgaris*, *C. minutus*, *C. reinhardtii*, *M. aeruginosa*, *Synechocystis* sp.; prolate spheroid/ellipsoidal: *A. obliquus*, *O. marsonii*, *C. ovata*; coenobia with prolate spheroid/ellipsoid cells: *D. armatus*) and belong to the taxonomic class of chlorophytes, cyanobacteria and cryptophytes. For each species, different life cycle stages were investigated. Most species at stationary phase have a different phenotypic appearance. Some species show a high accumulation of large intracellular granula of reserve material (e.g. starch) (*A. obliquus*, *C. reinhardtii*, *O. marssonii*), encystment (*C. minutus*) or an increase in cell size (*M. aeruginosa*, *Synechocystis* sp.). Based on morphological similarities, derived from brightfield images alone it was expected that confusion between *A. obliquus*, *C. reinhardtii*, *C. vulgaris* and *O. marsonii*, *Synechocystis* sp. as well as between *C. minutus* and *M. aeruginosa* could occur.

### Identification on species level

**Fig. 4:**
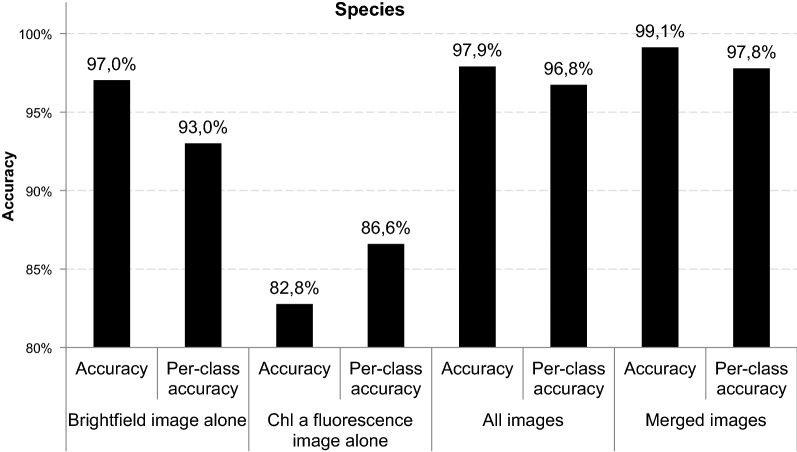
Accuracy and per-class accuracy as metrics for four different classifiers (1) brightfield images alone, (2) Chl *a* fluorescence images alone, (3) all brightfield—Chl *a* fluorescence images and (4) merged brightfield—Chl *a* fluorescence images to predict species identity

**Fig. 5:**
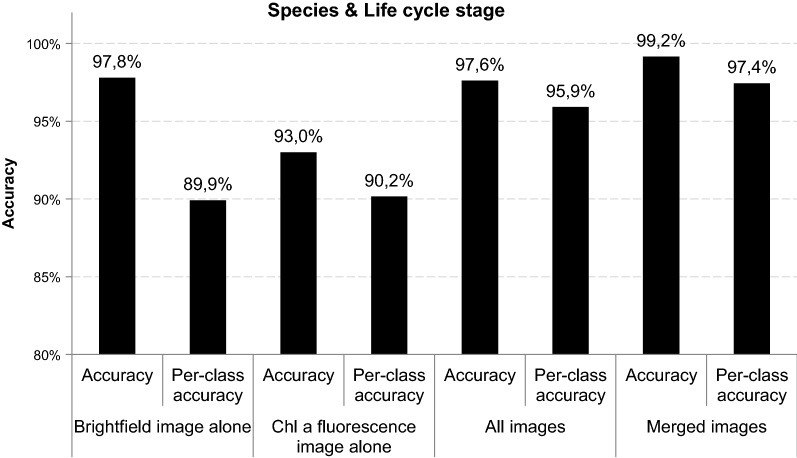
Accuracy and per-class accuracy as metrics for four different classifiers (1) brightfield images alone, (2) Chl *a* fluorescence images alone, (3) all brightfield—Chl *a* fluorescence images and (4) merged brightfield—Chl *a* fluorescence images to predict species identity and life cycle stage

The performance of the four different classifiers was evaluated in terms of accuracy and per-class-accuracy. Accuracy in Figs. 4 and 5 refers to the amount of all correctly classified

images in the test set, while averaged per-class accuracy indicates how well individual classes can be distinguished despite their imbalanced representation in our dataset. At the species level (Fig. 4), Chl *a* fluorescence images alone show the lowest overall accuracy with 83% and per-class accuracy with 87%, while combined images allow for the highest accuracy with 99% and a per-class accuracy of 98%. Interestingly, even brightfield images alone result in accuracy and per-class accuracy of 97 and 93% respectively.

**Fig. 6:**
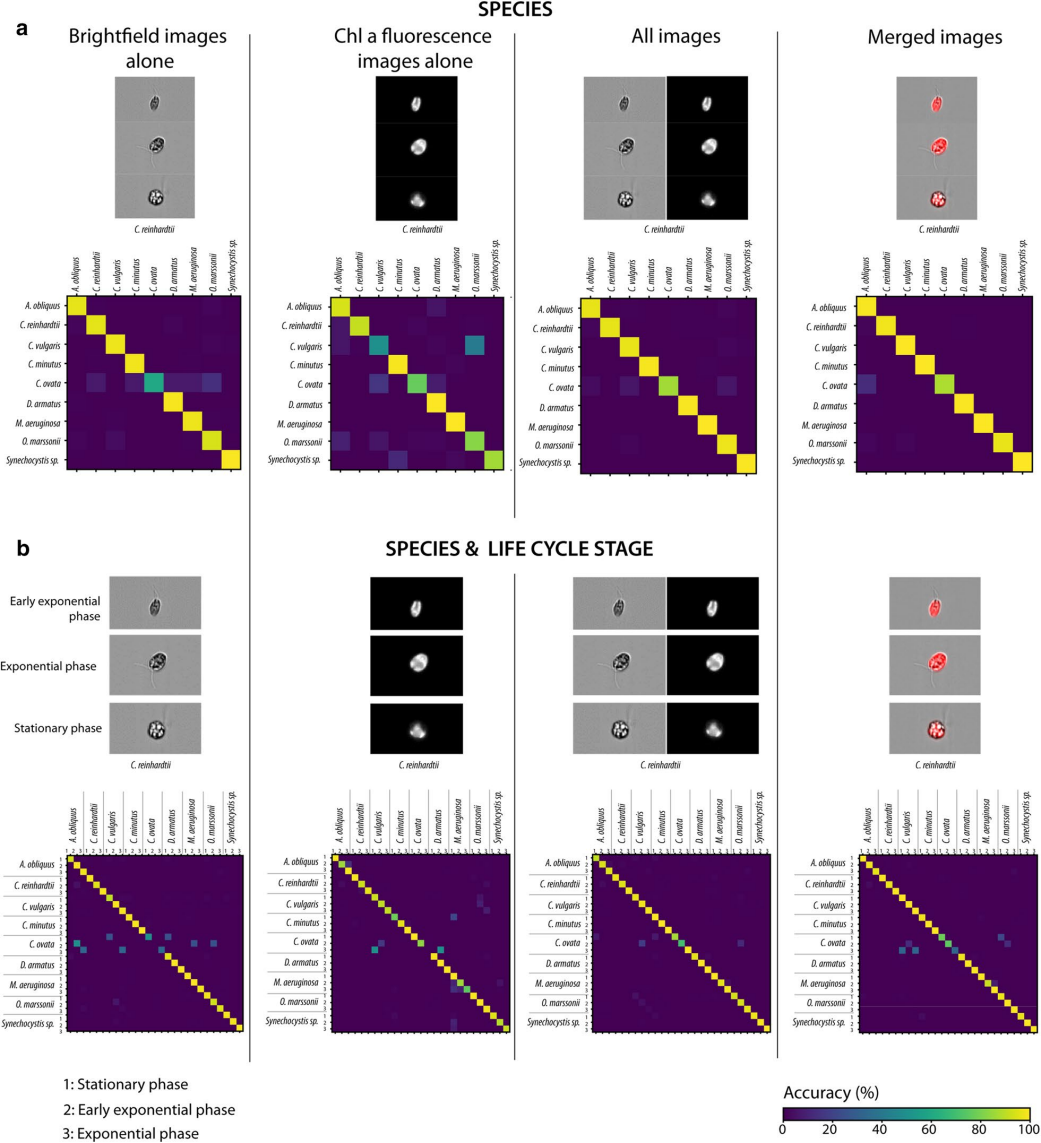
Exemplary images and confusion matrices for (**a**) species (**b**) and species and life cycle stage of four different classifiers trained on (1) brightfield images alone, (2) Chl *a* fluorescence images alone, (3) all brightfield—Chl *a* fluorescence images (“All images”) and (4) merged brightfield-Chl *a* fluorescence images (“Merged images”) solely identifying species and identifying species at different life cycle stages. The scale of the confusion matrices indicates the percentage of correct and incorrect classifications

Species dependent misclassifications were visualized in more detail, in form of confusion matrices shown in Fig. 6. This figure illustrates the instances of an observed species in rows versus the instances of the same species being predicted in columns. We present one matrix per image type, visualizing how a certain species was confused with others, if its accuracy was below 100% [43]. This is, for example, the case for *C. ovata* vs. *O. marssonii* for brightfield images. Using brightfield and Chl *a* fluorescence images alone increases the tendency towards false classification, while the classifier with merged images decreases false classification. *C. ovata* achieved the lowest classification accuracy across all species (Fig. 6) and was often misclassified as *O. marssonii* for brigthfield images and *C. vulgaris* for Chl *a* images. For the fourth category (merged images) *C. ovata* was confused with *A. obliquus.* Frequent confusion of *C. ovata* with green algae is on the one hand due to the fact that *C. ovata* is the least represented specie in the dataset (only 270 images could be provided for training) in contrast to all other species. On the other hand, Chl *a* fluorescence of *C. ovata* is highly variable and shows high similarity in intensity to green algae when not growing in the exponential phase (Fig. 1).

### Identification on species level & life cycle stage

Classification of life cycle stage in combination with species identification shows a similar picture as species identification alone (Fig. 5). The classifier performance improved by including both, brightfield and Chl *a* fluorescence images while Chl *a* fluorescence images alone yield the lowest accuracy. The classifier with highest accuracy and per-class accuracy is trained on merged brightfield and Chl *a* images.

Misclassification for species and life cycle stage as visualized in Fig. 6 for all classifiers most frequently occurred in *C. ovata* vs. green algae. In general, most misclassifications were detected when brightfield or Chl *a* fluorescence images were trained separately. Despite misclassification in *C. ovata*, Chl *a* images alone led to misclassification of *C. vulgaris* and *O. marssonii*, as well as misclassification of different life cycle stages of *M. aeruginosa*. A combination of images, either by taking all images or merged images into account, improved classification in a way that high misclassification rates only occurred for *C. ovata* vs. *C. vulgaris* and *O. marssonii*.

## Discussion

Phytoplankton species identification is a crucial part of water quality monitoring. But adequate monitoring is strongly limited by laborious microscopic techniques [17, 18].

Analytical flow cytometry can only partly be used to overcome the limitations of manual microscopy due to a large mismatch of taxonomic requirements and the highly compressed flow of cytometric single value outputs [8, 27]. Shallow-learning artificial neural networks and other traditional classification techniques were nonetheless successfully applied to distinguish species based on their scattering properties and fluorescence emission signals [1, 5, 6, 19, 50, 51]. Comparing accuracy of these approaches is complicated, because the size of datasets, the number of included parameters, the instruments and the image quality vary strongly among studies (cp. Table 2). Depending on the number of species and parameters considered, identification accuracy was between 70 and 98%. Nevertheless, the utilized training datasets consisting of scatter and fluorescence signals of laboratory grown cultures (e.g. [1, 6] are not representative for natural field samples [50]).

**Table 2:** Literature review for automated species identification for phytoplankton species identification with machine learning (neural networks), with flow cytometric data or images

	Reference	Network	Species/classes	Species size	Parameters included	Type of parameter	Instrument	Accuracy
Flow cytometric data (scatter and fluorescence values)	Frankel et al. [19]	ANN (Kohonen network/Back-propagation neural networks)	5	Picoplankton, large phytoplankton	5	FSC 488 nm Ex./540–630 nm 514 nm Ex./540–630 nm 488 nm Ex./660–700 nm 514 nm Ex./660–700 nm	EPICS V	92–100%
	Balfoort et al. [1]	Multilayer feedforward network (NWorks, ANNET, 8)	8	3–3500 µm	6	FSC, SSC, TOF 488 nm Ex./515–600 nm Em 488 nm Ex./650–750 nm Em 633 nm Ex./650–750 nm Em	Optical Plankton Analyzer	90–98%
	Boddy et al. [6]	Back-propagation neural networks, hierarchical approach	40	3–40 µm	6	FSC (horizontal, vertical), SSC, TOF 488 nm Ex./> 660 nm 488 nm Ex./530–590 nm	EPICS 741	> 70%
	Wilkins et al. [51]	MLP, RBF	42	Boddy et al. [6]				68–74%
	Wilkins et al. [52]	RBF ANN	34	3–1000 µm	11	FSC, SSC, TOF 488 nm Ex./Red Em 488 nm Ex./Orange Em 488 nm Ex./Green Em 630 nm Ex./Red Em Diffraction module: Vertical bar, horizontal bar, outer ring, inner ring	EurOPA	92%
	Boddy et al. [5]	RBF NN	72	1–45 µm	7	FSC-H, SSC-H, FL1-H, FL2-H, FL3-H, FL3-A, TOF	FACSort™	70–77%
Pulse shape	Malkassian et al. [33]		20	n. a.	8	FSC, SSC FLR (668–734 nm Em.) FLO (601–668 nm Em.) FLY (536–601 nm Em.) Pulse shape descriptors (shape, length and area under the pulse)	CytoSub	78%
Images	Gorsky et al. [23]		3	3–43 µm	5	Area, Circularity, Convexity, Length, Perimeter	Autonomous Image Analyzer/HIAC	–
	Embleton et al. [17]	MLP	4	10–390 µm	74	Area, Circularity, Diameter, Fibrelength, Grey level values (SD, Skewness, Kurtosis), Perimeter	Microscope camera (Sony DXC-930P)	67–93%
	Sosik and Olson [45]	SVM	15 (natural samples)	10–100 µm	22 categories/210 elements	Size, shape, symmetry, Texture characteristics, Diffraction, Co-occurence	FlowCytobot	68–99%
	Blaschko et al. [4]	SVM	13 classes	n. a.	780 features	Simple shape, moments, contour, differential, texture	FlowCam	71%
	Correa et al. [9]	CNN	19 classes	n.a.	–	–	FlowCam	89%
	Rodenacker et al. [42]	DT, LDA	23	n. a.	5	Shape, significant points, principal components, contour, fourier descriptor, extinction, Shape moments, colorimetry, fluorimetry	Inverse microscope	76%
	Chen et al. [8]	CNN, PCA + SVM	1	n. a.	16	Diameter, tight area, perimeter, circularity, major axis, orientation, loose area, median radius, opd, refractive index, absorption, scattering	TS-QPI	< 85%
	Li et al. [31]	CNN	9	n. a.	–	–	Mueller matrix microscope	97%
	Pedraza et al. [37]	CNN	80	n. a.	–	–	Microscopy	99%
	This study	CNN	9	1–90 µm	–	–	ImageStream^®X^ MK II	97%

The respective network, species or classes, species size, parameter number and parameter type, instruments or technique and the respective accuracy is provided. For some studies it was not possible to get details from the text about investigated cell size range

*TOF* Time of flight, *MLP* Multilayer Perceptron/backpropagation network, *RBF* radial basis function, *SVM* support vector machine, *TS-QPI* Time stretch quantitative phase imaging, *OPD* optical path length difference, *n. a.* not available, *Picoplankton* 0.2–2 µm, *Nanoplankton* 2–20 µm, *Microplankton* 20–200 µm, *Macroplankton* 200–2000 µm

We argue that in contrast, image-based training datasets are more robust and suitable to reflect the large range of environmental conditions and mimick these conditions through different batch culture stages in this study. Gorsky et al. [23] pioneered research in this area using microscopic images and a set of simple geometric features to distinguish between three species (*Prorocentrum micans*, *Nitzschia closterium* and *Hymenomonas elongata*) of distinct size and shape. Blaschko et al. [4] achieved 50–70% classification accuracy with FlowCam images depicting twelve phytoplankton classes (centric diatoms, pennate diatoms, dinoflagellates, ciliates, unidentified cell, non-cell, *Mesodinium*, *Laboea*, *Skeletonema*, *Thallasiosira*, *Thallasionema cf.*, *Pseudo*-*nitzschia*) and an “unknown” class using shape features, texture features, and contour. In the study by Blaschko et al. species were measured at a magnification of 4× or 10× and the cell size spectrum was between ~ 10–60 µm. Rodenacker et al. [42] applied fluorescence imaging in their image acquisition system with a magnification of 20× and 40× to capture more information for discrimination between five classes of phytoplankton (*Peridinium umbonatum*, *Cryptomonas erosa*, *Cryptomonas marsonii*, *Trachelomonas* sp. and *Ankistrodesmus* sp.). The authors used species with a cell size in the range of 10–30 µm and report 58–93% correct identifications. Sosik and Olson [45] used the FlowCytobot and a combination of image features including size, shape, symmetry, and texture characteristics to train a support vector machine (SVM) classifier (SVM classifier is a supervised learning model with associated learning algorithms) identifying 22 species (*Asterionellopsis*, *Chaetoceros*, *Cylindrotheca*, *Cerataulina* spp.+ *Dactyliosolen* similar to *Cerataulina* spp. (DactFragCeratul), other *Dactyliosolen*, *Dinobryon*, *Ditylum*, *Euglena*, *Guinardia*, *Licmophora*, *Phaeocystis*, *Pleurosigma*, *Pseudonitzschia*, *Rhizosolenia*, *Skeletonema*, *Thallasiosira*, ciliate, detritus, dino, flagellate, other < 20 µm and pennate) with an accuracy of 88%. The study by Sosik and Olson used a species range between ~ 5–400 µm, where most species were in a range between 20 and 50 µm. Schulze et al. [44] trained a traditional shallow-learning neural network classifier based on shape, texture and fluorescent features from microscopy phytoplankton images and reported a classification accuracy of 94.7% for ten taxa (*Cyclotella menighiana*, *Anabaena* sp., *Chlorogonium elongatum*, *Cryptomonas ovata*, *Desmodesmus perforates*, *Staurastrum tetracerum*, *Botryococcus braunii*, *Pediastrum duplex*, *Trachelomonas volvocina*, *Crucigenia tetrapedia*), a comparable cell size spectrum to this study. The studies by Blaschko et al. [4], Rodenacker et al. [42], Sosik and Olson [45] and Correa et al. [9] used species with a comparable large cell size, while this study should explore the lower edge of cell size range. We hypothesize that when small species can be well distinguished, larger species will be less problematic due to more detailed morphological structures.

In this study, we demonstrated a very high accuracy in species identification (99% accuracy, per-class accuracy 97.8%) with nine comparable small species by directly analyzing brightfield images (morphological information) and Chl *a* fluorescence images (chloroplast morphology and Chl *a* fluorescence intensity) in combination rather than the highly compressed multi-variate scatter and fluorescence emission signals. Even when solely classifying based on brightfield images, the classifier delivered respectable accuracy (97% accuracy, 93% per-class accuracy). A similar high accuracy was reached by Li et al. [31] (97%) and Pedraza et al. [37] (99%) by the use of microscopic images in combination with CNN-training. Both authors did not use an imaging flow cytometer to collect the images, but used semi-automated microscopic systems. The high automation and in best case one-cell-at-once-analysis of the system used in this study is a major advantage for future phytoplankton analysis. Authors using other imaging flow cytometers (FlowCytobot, FlowCam) used SVM networks and reached accuracy of 68–99% [45] and 71% [4]. The question is whether accuracy of image recognition achieved on images taken with the Flowcytobot or the FlowCAM could profit from CNNs. Here Correa et al. [9] could show 89% accuracy for FlowCAM images by using a CNN approach with 8 layers. An additional point for difficulties in comparison with the current study is the point that Sosik and Olson [45], Blaschko et al. [4] and Correa et al. [9] used phytoplankton images from field measurements with more artifacts and trash. In future studies the applicability of the system used in this study shall be investigated for field measurements. The 60× magnification was demonstrated as suitable magnification to collect images of cells in the size range of 1–90 µm, representing a crucial part of natural phytoplankton communities [45]. For larger genera (data not shown), e.g. *Asterionella*, *Anabaena* or *Planktothrix* the 20× magnification would be more useful due to a larger field of view.

It would in theory be possible to include additional fluorescence images from different excitation and emission channels, but the accuracy could be only marginally improved, because it was already really high. However, additional image channels may further increase the robustness of species identification and especially for future issues dealing with higher complexity in natural samples it could be relevant to include images from additional fluorescence channels. This needs to be evaluated with a larger dataset, containing more taxonomic groups and species. The extra channels are nevertheless relevant for taxonomic pre-sorting of data by assigning larger taxonomic groups and to subsequently apply a deep learning approach identifying species for these specific taxonomic groups. A balanced number of images (the same number of images for each species) for training would be ideal, to better compare classifier accuracy. Alternatively, per-class accuracy was established as comparative parameter, because all images collected should be used for training.

In this study, we took as additional prediction parameter ‘life cycle stage’ into account. Batch cultivation, starting with a small inoculum, a lag, early exponential, exponential and stationary phase is a good way to mimic different life cycle stages, which could also occur under field conditions. It is expected that the physiological performance varies under these different cultivation phases, whereby it is highest during exponential phase and lowest during stationary phase. Especially the stationary phase is a phase of high nutrient and light deficiency. Like described in other studies as well, cells are able to prepare for long phases of deficiency by accumulating storage compounds, like starch grains or cyanophycin [15, 16, 40]. In our study, we found starch grain like granules in stationary phase cells of *A. obliquus*, *C. reinhardtii*, *D. armatus*, and *O. marssonii*. An additional indication of cellular adaption to nutrient deficiency is a large cell size [15], like observed for all species with an accumulation of starch grains and *C. vulgaris*, *M. aeruginosa* and *Synechocystis* sp. Long et al. [32] suggest cell size increase as a good stress indicator. For *C. minutus* a thick wall layer could be identified from the images as kind of encystment, being an adaption to long term persistence, like described by Ellegaard and Ribeiro [16].

In addition to species identification alone, estimation of life cycle stage, nutrient deficiency or much more general a stressor could be a valuable feature for biotechnological applications or field studies by informing about ongoing primary production or grazing effects. Furthermore, evidence for a mixing event or the end of the vegetation period could be concluded if encysted, enlarged or storage material containing cells are detected in the water body. In biotechnological approaches life cycle stage monitoring could be valuable for process control.

Deep learning artificial neural networks automate the otherwise subjective critical feature extraction step by learning a suitable representation of the training data and by systematically developing a robust classification model. In contrast to existing approaches there is a higher similarity to taxonomic approaches by considering detailed morphological information as well as chloroplast morphology, color and intensity. Deep learning approaches show remarkable performance, but the process behind is kind of a black box and transformation into white-box is in the early stages [34]. It is important to be aware of artifacts, like probably occurring in the background of the phytoplankton images, taken with the imaging flow cytometer. To consider and exclude potential influence of background on species identification, we carefully augmented images during training, in terms of brightness, saturation, contrast and hue-values, to induce variations of the image background. In comparison to other image recognition tasks, e.g. with images from natural landscapes, the background in images from an imaging flow cytometer used is much more homogeneous and stable, because optical settings are calibrated each day before the start of the measurements.

The case of *C. ovata* shows that 20–63 images per class were not sufficient to train the network sufficiently. In contrast, *D. armatus* in early exponential phase had only 235 images available, but is properly classified. Therefore it is suggested to collect at least 200 images in order to develop a robust species recognition classifier. However, further evaluation in this direction is required since the minimum number of training images may also be species-dependent, for example species with special characteristics may require less images. If sufficient images are available, it can be expected that the learning curve of machine learning approaches is more predictable [25] than human learning curve and can be done in shorter time, due to continuous training. By using one classifier for species identification, much higher objectivity is given. However, even though there is doubt about correct identification, automatic image recording allows archiving data and to perform a subsequent proof of identification at a later point in time [42].

The approach suggested in this study should be extended to a larger number of species, but could then be helpful for many operators, responsible for phytoplankton monitoring, e.g. within the European Water Framework Directive, Great Lakes Phytoplankton Monitoring by the Environmental Protection Agency of the United States (EPA) or the monitoring program of ballast water of the International Maritime Organization. The method enables the operator to get an automated instantaneous, archivable, objective and quantitative information about water quality relevant phytoplankton communities.

## Conclusion

There are reasonable doubts concerning the reliability of AFC for indicator-taxa identification due to low taxonomic resolution, but these concerns may be overcome given there is a microscopic image for each single measured cell.

In this study, we presented for the first time an automated approach for identifying species and their life cycle stage utilizing state of the art machine learning techniques (CNNs), working best when using a classifier trained on merged brightfield and Chl *a* fluorescence images. Species identity and in addition life cycle stage have been predicted successfully, while flow cytometric measurements provide quantitative data for each single species.

Our study is an initial milestone for future improvement, e.g. with a larger set of species. The configuration of the image-based cytometer used, would also allow to cover a broader phytoplankton size spectrum (cells up to 100 µm width, when arranged along the fluid stream in longest axial dimension) by taking images at lower magnifications (20× or 40×). In addition, the robustness of classifications could be further improved by pooling different measurements taken at different times and environmental conditions, as well adding additional fluorescence images. In this study, we demonstrated that a CNN classifier can recognize species, even if their Chl *a* fluorescence pattern was extremely different, depending on the respective life-cycle stage.

Manifold necessary monitoring tasks, like ballast water monitoring and other national and international monitoring programs, could potentially profit from the suggested combination of high through-put imaging flow cytometry and deep learning. In future, a detailed evaluation of the method against traditional microscopic species identification is needed.

## Abbreviations

AFC: analytical flow cytometry
ANN: artificial neural networks
Chl *a*: chlorophyll *a*
CNN: convolutional neural networks
FCM: flow cytometry
FSC: Forward scatter signal
IFC: imaging flow cytometry
SSC: Sideward scatter signal
SVM: support vector machine
